# Beyond raw comparisons: Adjusted analysis reveals only minor inter-hospital differences in ACDF outcomes in Norway

**DOI:** 10.1016/j.bas.2026.105976

**Published:** 2026-02-16

**Authors:** David A.T. Werner, Cecilia I.A. Avellan

**Affiliations:** Department of Neurosurgery Stavanger University Hospital, Norway

**Keywords:** Anterior cervical discectomy and fusion (ACDF), Patient-reported outcomes, Risk adjustment, Hospital benchmarking, Cervical radiculopathy, Multicenter registry study

## Abstract

**Introduction:**

Annual reports from the Norwegian Registry for Spine Surgery (NORspine) suggest notable inter-hospital variation in patient-reported outcomes after anterior cervical discectomy and fusion (ACDF) for radiculopathy.

**Resarch question:**

Do unadjusted comparisons not account for differences in case mix between institutions?

**Material and methods:**

This multicenter observational cohort study analyzed data from 7832 patients undergoing ACDF for cervical radiculopathy in Norway between 2014 and 2023. Risk factors for poor outcome were identified by multivariate logistic regression. Outcomes were measured using the Neck Disability Index (NDI), Numeric Rating Scale (NRS) for arm pain, and EQ-5D-5L at 12 months. The proportion of non-successful outcomes for five public hospitals and the private sector was adjusted for patient demographics and risk factors.

**Results:**

Unadjusted, the range of non-successful outcomes between hospitals was up to 25%. After risk adjustment, inter-hospital differences diminished markedly (range 1.9%–3.6%). Only private sector status remained statistically significant in all three models. High odds ratios (>2.0) for non-success were observed for medical litigation, prior cervical surgery, duration of arm pain >12 months, and headache as a dominant symptom.

**Discussion and conclusion:**

Apparent differences in ACDF outcomes across Norwegian hospitals were largely explained by patient-related risk factors. Adjusted analyses revealed only minor inter-hospital variations. For registry-based quality benchmarking, risk-adjusted reporting is essential to support transparent comparison and informed clinical decision-making.

## Highlights

• Logistic regression analysis revealed several predictors of poor outcome 12 months after ACDF surgery.
• Risk factor adjusted analysis of outcome data reduces variation between hospitals significantly.
• Quality registries should publish both adjusted and unadjusted data to enable transparent hospital comparisons.

## 1 Introduction

Anterior cervical discectomy and fusion (ACDF) is the most commonly performed surgery for cervical radiculopathy refractory to conservative treatment, with the numbers of surgeries constantly increasing in Norway (Kristiansen et al., 2016). With increasing availability of MRI diagnostics, the rate of surgical treatment is on the rise (Danielsen et al., 2022). In Norway six public hospitals and eight private hospitals perform ACDF procedures as of 2025. The country has government funded universal healthcare, and public hospitals are the primary supplier of specialized health care. The choice of public hospital depends on the patient's postcode, with the hospital assigned based on the patients' residential address, while private hospitals are accessible depending on the patient's individual insurance or means of direct reimbursement. In recent years challenges with treatment capacity and long waiting times have been a driving force towards private healthcare (Danielsen et al., 2022).

All hospitals performing surgery for degenerative disorders of the cervical spine report to the Norwegian Registry for Spine Surgery (NORspine), where data on indication, image findings, surgery, patient demographics, complications and patient reported outcomes (PROMs) are collected. The completeness rate for 2020 was 82%. Patient participation is voluntary, requires informed consent and does not influence the access to surgical treatment or the type of procedure performed. The registry is publicly funded and operates independently from the interests of the industry or individual medical centers (Mikkelsen et al., 2023).

Since 2010 the NORSpine registry publishes annual reports including national quality indicators with benchmarks, intending to enable comparison of results between hospitals and against aggregated national figures. These reports are meant to allow insights into trends and variations, such as the rising incidence of ACDF procedures and considerable geographic variations in surgical rates across Norway (Mikkelsen et al., 2023; NORspine Nasjonalt Kvalitetsregister for Ryggkirurgi). Furthermore, they are supposed to give insights into healthcare disparities between individual centers, based on established outcome criteria (McNeil et al., 2010). These reports, publicly accessible, seem to indicate considerable differences in outcomes and quality of care between the different public and private centers in Norway, measured at one year after the ACDF procedure (Solberg et al., 2024). These findings are valuable for administrators, policy makers and patients themselves, and have also garnered the attention of the media.

Two main observations are seemingly made based on the reports. First, private hospitals seem to outperform the public hospitals. Secondly, there are marked differences between the different public hospitals (Solberg et al., 2024). It is important to note that these observations are directly deducted from the way data is presented in the NORspine summaries. In consequence, patients in clinical practice draw conclusions regarding quality variations and request referrals to hospitals that benchmark better in the annual reports.

In Norway only very few cervical arthroplasties are performed, and nearly all ACDF procedures are done with an interbody fusion implant (PEEK or titanium), without additional plating or anchoring of the implant (Lied et al., 2010; Yoon et al., 2017). While the surgical procedure is rather uniform, the indication for ACDF in cervical radiculopathy is relative and depending on surgeon and patient preference. The question thus must be asked if the apparent quality variations published in the annual reports reflect differences in surgical aptitude or are caused by an inherent bias introduced by patient demographics and risk factors (selection bias).

The aim of this study is to analyze data from the NORspine and evaluate if the differences in patient reported outcome persist when adjusted for potential variations in patient demographics and the presence of established risk factors for outcome after ACDF procedures.

## 2 Materials and methods

### 2.1 Design

This a multicenter observational cohort study in adherence to the STROBE criteria and the methodological framework of the PROGRESS group (Steyerberg et al., 2013; Vandenbroucke et al., 2007).

### 2.2 Data source

Patient data from the NORspine were analyzed for the period of January 2014 to April 2023. The registry includes all patients operated in public or private hospitals for degenerative disorders in the spine, on a consent basis. In 2023, 84% of all cervical spine surgeries were reported to the registry (Solberg et al., 2024).

### 2.3 Ethics

Human Ethics and Consent to Participate declarations: not applicable. All participants provided informed consent prior to inclusion in the study via the NORspine questionnaire.

The study was conducted in accordance with the ethical principles of the Declaration of Helsinki as adopted by the World Medical Association (Bausch and Mandell, 1989). Approval for the study protocol was obtained from the internal review board for Stavanger University Hospital (SUS Pasientvernombud reference number 4662) and the Norwegian Directorate of Health has granted an exemption from the duty of confidentiality for use of data from the NORspine (Helsedirektoratet 24/1042-6). The Norwegian Data Inspectorate has approved the overall NORspine protocol.

### Funding

No funding has been received in conjunction with this study.

### 2.4 Eligibility criteria

Patients under the age of 16, or suffering from severe psychiatric disorders, drug abuse or cognitive impairment are excluded from the registry. For this study only patients operated for cervical radiculopathy were selected. Patients operated with ACDF for other conditions (i.e. cervical myelopathy) were excluded from the analyses.

### 2.5 Measurements

When admitted for surgery patients fill in a baseline questionnaire, collecting data on demographics, lifestyle factors and PROMs. At time of surgery the surgeon registers data on indication, image findings, surgical procedure and complications. At both 3- and 12 months after surgery a questionnaire containing PROMs and the general perceived effectiveness (GPE) of the intervention is answered by the patients.

### 2.6 Patient reported outcome measures

For cervical surgery the NORspine utilizes three PROMs, the Neck Disability Index (NDI), Numeric Rating Scale (NRS) arm pain and neck pain, and the European Quality of Life – 5 Dimensions questionnaire (EQ-5D-5L) (Mikkelsen et al., 2023). The NDI assesses the impact of neck pain on the quality of life in several dimensions (pain, personal care, lifting, reading, headache, concentration, work driving, recreation and sleeping). The disability is scored for each dimension on a 6-point scale (0 = no disability, 5 maximal disability), and a total score is calculated between 0 and 100 points where 100 is the maximal amount of disability. The NDI is considered a validated and reliable instrument when assessing outcome after degenerative spine surgery in the neck, and binary outcome cutoffs have previously been defined as goals to be reached for the surgery to be considered success (Mjåset et al., 2020, 2022a, 2022b)–(Mjåset et al., 2020, 2022a, 2022b).

The NRS is a widely used self-reported measure of pain severity using a scale from 0 (no pain) to 10 (worst conceivable pain). In the NORspine both neck pain and arm pain before and after surgery are assessed by the NRS.

The EuroQol-5D-5L (EQ-5D-5L) is a standardized instrument for measuring health-related quality of life, assessing five dimensions: mobility, self-care, usual activities, pain/discomfort, and anxiety/depression, each with five severity levels (Herdman et al., 2011).

Based on previous studies and the benchmark criteria of the NORspine, the outcome criteria for a negative outcome one year after ACDF (non-success) are an NDI percentage change of less than 35%, an NDI absolute score above 26, or a NRS score of arm pain above 3, respectively (Mikkelsen et al., 2023; Mjåset et al., 2022a).

### 2.7 Hospitals

In Norway six university hospitals and eight private hospitals have reported to the NORspine during the study period. Due to their lower individual case numbers, the private hospitals are summarized for the purpose of this analysis.

### 2.8 Statistical analyses

All analyses were performed with the Statistical Package for Social Sciences (SPSS, version 23, IBM) and Excel (Microsoft). To identify risk factors for the three different outcome classifiers, multivariate logistic regression was utilized. Missing values were treated as random. Based on previous literature, the following potential predictive factors were included in the univariate analyses; Female gender, Age >60, employment status, educational level, type of physical occupation (computer/desk, light work, hard physical work), nonnative speaker, enrolled in medical litigation, smoking, having undergone previous cervical surgery, undergoing surgery >1 cervical levels, arm pain duration (<3 months, 3-12 months, >12 months), BMI >30, comorbidity, diabetes mellitus type 2, ASA >2, NRS neck pain worse than NRS arm pain, preoperative NDI levels (<40, 40-60, >60), NDI headache categories, preoperative NRS arm levels (0-5, 6-7, >8), scoring for anxiety or depression on the EQ-5D for, and being treated in a private institute. Previously reported risk factors were dichotomized (Age, BMI, ASA grade etc.) and odds ratios were calculated. The hospitals were evaluated by the deviation function in the regression model, evaluating each unit against the average of all units. Collinearity between possible risk factors was assessed by Pearson correlation, with coefficients classified as weak (>0.3), moderate (>0.5), and strong (>0.7). Factors that were not significant in the multiple regression models were removed based on levels of significance (p < 0.05) in a backward fashion, except for the hospitals that were kept to adjust for each unit. The final models were assessed by bootstrapping five thousand samples for robust accuracy. From the regression coefficients of the final models’ probabilities were calculated. Six public hospitals and the private sector were compared individually. For each institution, the percentage of negative outcomes based on each of the three models was calculated (mean of the outcome). Consequently, the predicted probability for each unit based on the given case mix of the patient cohort, was subtracted from the mean of the cutoff variable. The resulting value represents the adjusted proportion of negative outcome. Positive adjusted values indicate a resulting performance worse than the model predicted and negative (minus) adjusted values a performance better than the model predicted.

## 3 Results

For this analysis, 7832 patients operated with ACDF for cervical radiculopathy between 2014 and 2023 were identified in the NORSpine registry. Of those 5005 patients (74%) had 12-month follow-up data available. Patient characteristics, surgical variables and patient reported outcomes are presented in Table 1.

**Table 1:** Patient data at baseline and at 12 months after surgery.

Variable	n	%
Responder 3 months	5757	73.5
Responder 12 months	5005	63.9
Female	4200	53.6
Age >60	1252	16
Smoker	2002	25.6
Lower education	6413	83.2
Non native speaker	697	8.9
Hard physical work	1839	23.5
BMI >30	1989	25.9
Other somatic illness	3572	45.6
ASA >2	666	8.5
Anxiety/Depression	523	6.7
Disability pension (incl applied for)	787	10
Litigation	579	7.4
Paresis	3818	76.3
Paresis duration >3 months	4169	75.3
Neck pain >3 months preop	6623	86.4
Neck pain >12 months preop	4170	54.4
Arm pain >3 months preop	6385	83.7
Arm pain >12 months preop	3336	43.7
NDI <40 preop	3856	52.2
NDI 40 - 60 preop	2868	36.6
NDI >60 preop	664	9
Compl. Paresis	702	15.7
Compl. Swallowing disorder	448	10
Compl. Vocal disorder	412	9.2

Variable	Mean	SD
NDI 12months	22.86	18.417
NDI improvement %	43.15	44.62

NRS arm pain 12months	2.78	2.83
NRS arm improvement %	52.51	56.2
NRS neck pain 12 months	3.21	2.76
NRS neck pain improvement %	43.09	60.53

Responder – has responded to the NORspine registry form. Lower education – less than 4 years of university. Hard physical work – not working at a desk or with light physical labor. Anxiety/Depression as scored on the EQ-5D-5L form. Litigation – enrolled in litigation against the Norwegian medical welfare fund. Paresis – as scored based on patients perception. Compl. – complication at 12 months post surgery, as reported by the patient. NDI – Neck Disability Index. NRS – numeric rating scale.

**Fig. 1:**
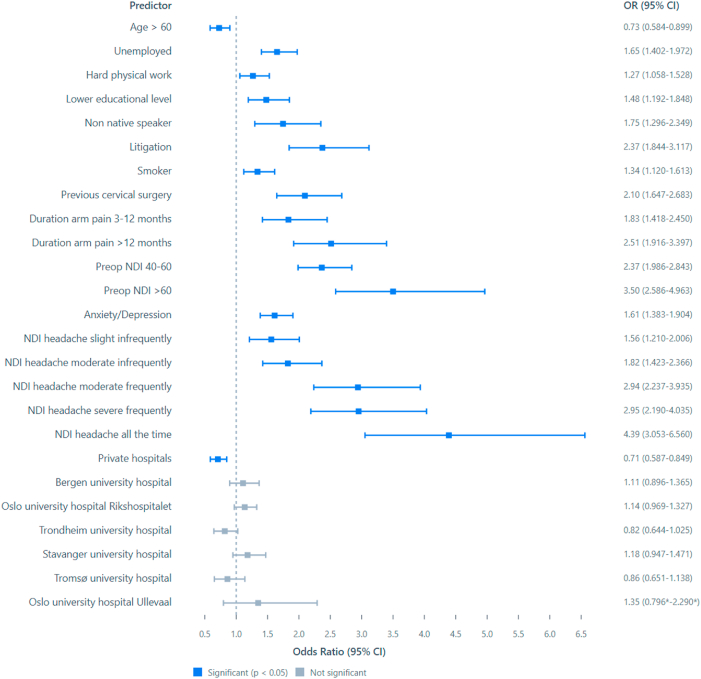
Forest plot of the binary logistic regression model predicting NDI raw score above 26 at 12 months after surgery. CI – 95% confidence intervals from the bootstrapped iterations. Values to the left of the stapled line indicate odds of positive outcome, values to the right odds for negative outcomes at 12 months after the surgery. ∗CI from the parametric analysis due to the deviation function in SPSS.

**Fig. 2:**
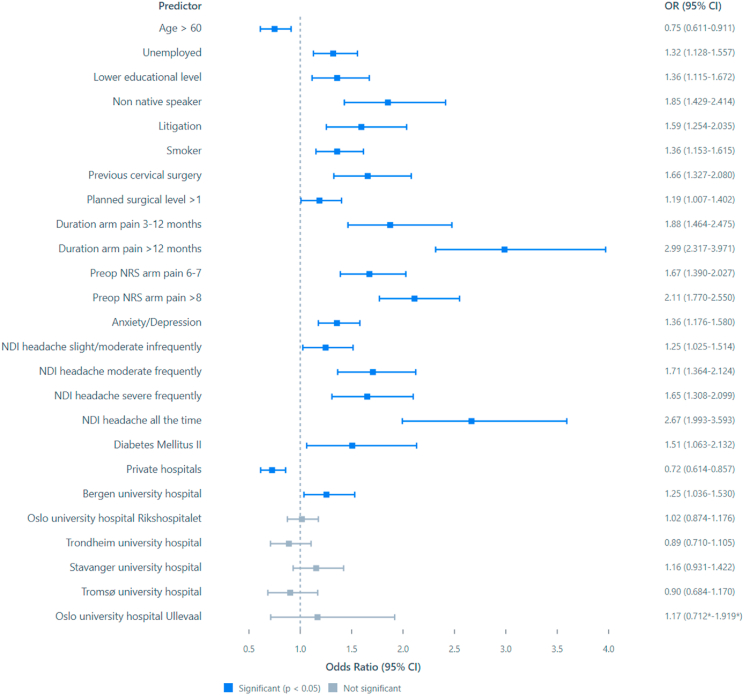
Forest plot of the binary logistic regression model predicting NRS arm score above 3 at 12 months after surgery. CI – 95% confidence intervals from the bootstrapped iterations. Values to the left of the stapled line indicate higher odds of positive outcome, values to the right higher odds for negative outcome at 12 months after the surgery. ∗CI from the parametric analysis due to the deviation function in SPSS.

**Fig. 3:**
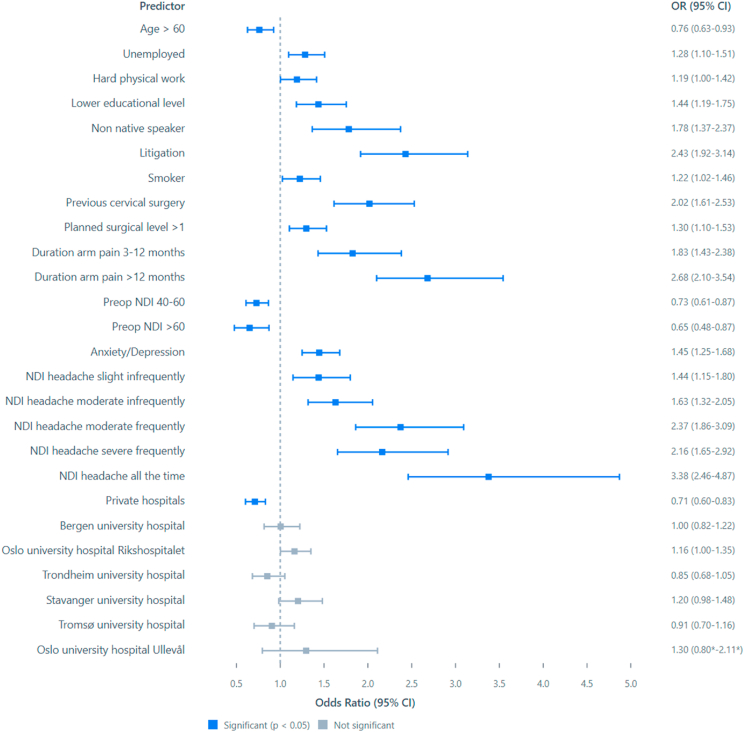
Forest plot of the binary logistic regression model predicting NDI improvement by less than 35% 12 months after surgery. CI – 95% confidence intervals from the bootstrapped iterations. Values to the left of the stapled line indicate higher odds of positive outcome, values to the right higher odds for negative outcome 12 months after the surgery. ∗CI from the parametric analysis due to the deviation function in SPSS.

The final multivariate model for all three models is shown in Fig. 1, Fig. 2, Fig. 3. For the tabular values, see the appendix. Being unemployed or having a physically demanding job, less than four years of university education, non-Norwegian mother language, being involved in medical litigation, smoking, previous cervical surgery, anxiety or depression, duration of arm pain above 3 months, as well has NDI headache severity were significant predictors of non-success shared in all three models. Educational level and job physicality correlated weakly (0.362), as well as NDI headache categories and overall NDI score (0.462). All other correlations were below 0.3.

**Fig. 4:**
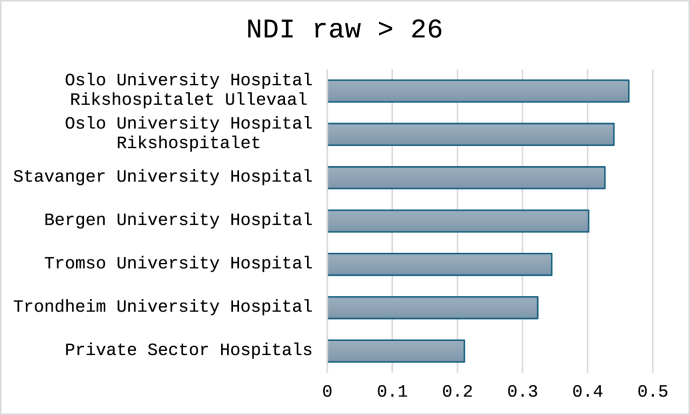
Proportion of non-successful outcome achieved by each hospital, before adjustment. Outcome criterion defined as NDI score >26, 12 months after surgery.

**Fig. 5:**
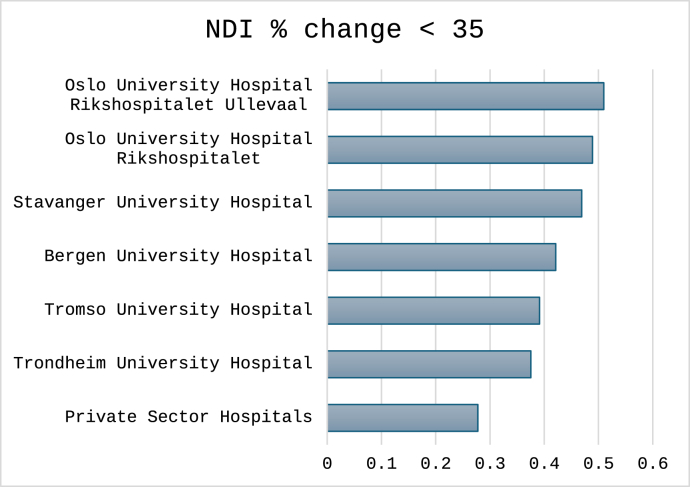
Proportion of non-successful outcome achieved by each hospital, before adjustment. Outcome criterion defined as NDI improvement <35%, 12 months after surgery.

**Fig. 6:**
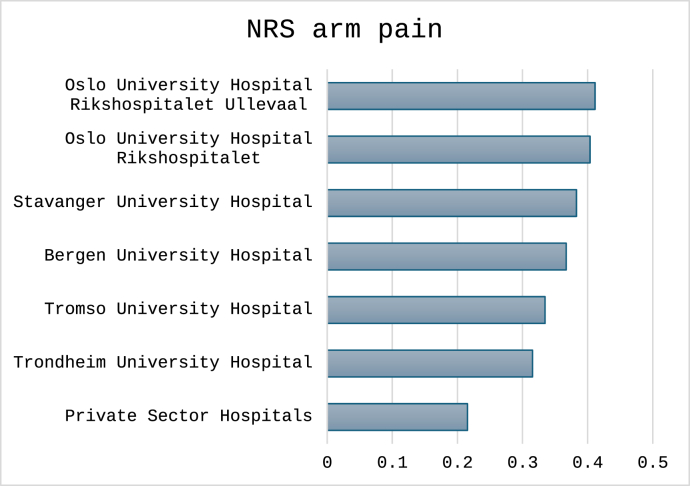
Proportion of non-successful outcome achieved by each hospital, before adjustment. Outcome criterion defined as NRS arms score >3, 12 months after surgery.

**Fig. 7:**
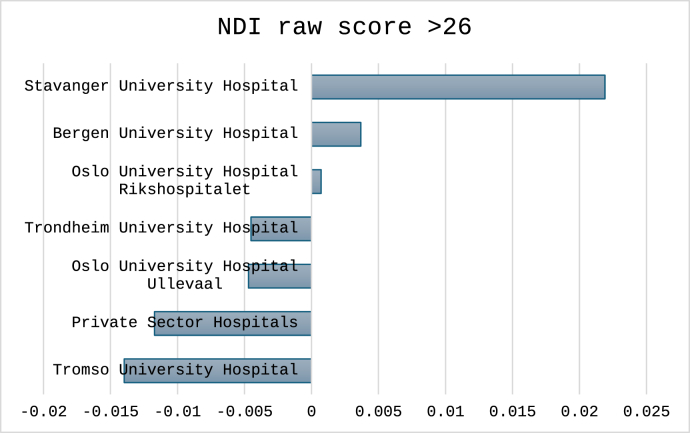
Adjusted outcome proportions achieved by each hospital. Outcome criterion NDI score >26, 12 months after surgery. For adjustment the modelled probability for the outcome was subtracted from the achieved outcome. Negative values indicated performance better than model prediction.

**Fig. 8:**
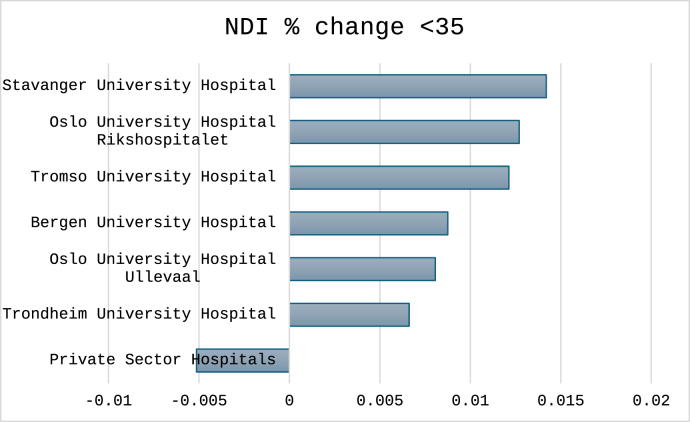
Adjusted outcome proportions achieved by each hospital. Outcome criterion NDI improvement <35%, 12 months after surgery. For adjustment the modelled probability for the outcome was subtracted from the achieved outcome. Negative values indicated performance better than model prediction.

**Fig. 9:**
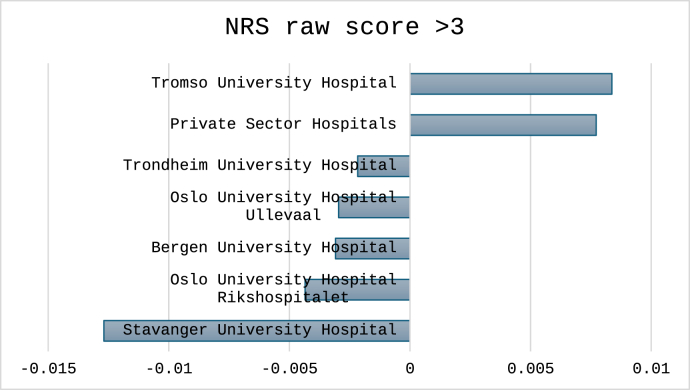
Adjusted outcome proportions achieved by each hospital. Outcome criterion NRS arm pain >3, 12 months after surgery. For adjustment the modelled probability for the outcome was subtracted from the achieved outcome. Negative values indicated performance better than model prediction.

For the three cutoffs classifying non-success, the total number (%) of cases were 1496 (30%) for NRS arm pain, 1712 (34%) for NDI percentage change and 1596 (32%) for NDI raw score. Outcomes classified for each hospital are shown in Fig. 4, Fig. 5, Fig. 6. In Fig. 7, Fig. 8, Fig. 9 the predicted probabilities have been subtracted from the outcomes, showing adjusted percentages based on the weight of predictive factors in the patient population treated in each hospital for the given period. Prior to adjustment the percentage of non-successful outcomes differed from highest to lowest by 25% (NDI raw), 20% (NRS arm), and 23% (NDI % change), indicating potentially significant difference in quality of care. After subtracting the probability for each outcome from the mean outcome for each hospital or the private sector, these differences were reduced to 3.6% (NDI raw), 2.1% (NRS arm) and 1.9% (NDI % change). Thus, differences in magnitude of non-successful outcomes were reduced by 86-92%.

When including the five public hospitals and the private sector in the regression models, only the private sector remained statistically significant at a p level <0.05 in all three models, as compared to running the model without adjusting for patient risk factors, where significant differences were only found for Bergen University Hospital and Oslo University Hospital Rikshospitalet (NDI raw outcome, and NRS arm pain raw outcome), and Oslo University Hospital Rikshospitalet and Stavanger University Hospital (NDI 35% change outcome) in addition to the private sector for all models. For model predicting NRS arm pain also Bergen University Hospital remained statistically significant after all adjustments.

## 4 Discussion

In this national Norwegian multicenter cohort study of patients operated with ACDF for cervical radiculopathy, a substantial proportion did not achieve successful outcome at 12 months when applying three different classifiers (arm pain cutoff, NDI cutoff, and percentage NDI change). Unadjusted NORspine data suggested up to a 25% difference in outcome between private and public hospitals, and up to 12% between public hospitals. However, if adjusted for the risk factors identified in the logistic regression models, the gap between the worst performing public hospital and the private sector was reduced to 2%. Moreover, in models using NDI and NRS arm pain cutoffs, private hospitals were no longer the best performers after adjustment. As these actors draw from a patient population with an advantageous risk profile, this potentially translates to poorer outcomes for the public hospital in the same catchment area based on the current presentation of data in the registry (Danielsen et al., 2022).

Reporting only unadjusted outcomes in NORspine may therefore mislead patients and policy makers. The American College of Surgeons National Surgical Quality Improvement Program (ACS NSQIP) recommends that quality registries should report risk adjusted data to inform on patient outcomes, allowing for meaningful comparisons between hospitals and surgeons who may serve dissimilar patient populations (ACS National Surgical Quality Improvement). Without these adjustments quality comparison can be misleading. This holds true for both spine surgery, but also for other clinical fields such as joint arthroplasty and even cancer treatment (Asher et al., 2022; Rolfson et al., 2016; Sibert et al., 2021; Sivaganesan et al., 2019). Risk outliers can furthermore be identified and addressed in quality improvement efforts (Krell et al., 2014)

We agree that risk adjustment can introduce bias, especially when reporting is done by individual surgeons within institutions. However, the large-scale data collection by an independent source such as the NORspine should reduce the risk of data manipulation (“gaming”) to affect outcome reporting (Siregar et al., 2013)

To accommodate different stakeholders, NORspine reports could adopt either a dual framework showing both adjusted and unadjusted outcomes with clear explanation of the impact of patient risk factors, or a stratified approach where outcomes are presented separately for distinct risk strata. Such formats would improve the interpretability of differences between hospitals and sectors and support more appropriate benchmarking.

This study is weakened by patient selection bias where almost one third of patients are non-responders at 12 months. In a previous study from NORspine this population has shown to have a slightly worse risk profile than responders, potentially skewing the outcome (Werner et al., 2017). In 2022 Ingebrigtsen et al. traced non-responders after cervical spine surgery and concluded that these patients were missing at random, and that respondent data is representative (Ingebrigtsen et al., 2023). Logistic regression has limitations when predicting outcome variables. Binary predictors might exclude relevant information from the analyses (Halicka et al., 2023; Osorio et al., 2016). While the overall accuracy was good, studies from the NORspine have shown that more advanced modelling such as machine learning can yield more accurate outcome predictions (Berg et al., 2024). However, the odds ratios and probabilities from logistic regression analyses allow for a transparent understanding and discussion of the importance of each risk factor, while machine learning models are more obscure in their approach. The effect of each individual factor on the outcome can also be translated to quality improvement efforts for each hospital, i.e. focus on reduced waiting times or patients that report high levels of headache vs. patients with significant comorbidities or high levels of previous operations at other hospitals (Sivaganesan et al., 2019).

This study draws strength from the real-life data of a large-scale clinical registry, collected in a prospective fashion. Identified risk factors are in concordance with reported literature, both for the NORspine and other clinical registries. Both private and public hospitals report to the registry, allowing for comparison between the sectors. Risk factors identified are consistent with previous Norwegian and international studies (Archer et al., 2020; Hébert et al., 2022; Mjåset et al., 2022a). Factors yielding particular high odds ratios can be explained by secondary benefit intention (litigation), previous non-successful surgery with potentially weak surgical indication (previous surgery), long symptom duration with pain becoming a chronic issue and patient exhaustion (duration of arm pain), and patient focus on symptoms that respond poorly to an anterior discectomy and fusion in the cervical spine (headache). Of note even when adjusting for baseline risk factors, undergoing surgery in a private institute was associated with a lower chance for non-successful outcome. This is also indicated by the outperformance of private practice versus predicted results in all three models and has been previously shown in a NORspine study (Danielsen et al., 2022).

## 5 Conclusion

The difference in outcomes between the public hospitals, and between public and private sector hospitals in Norway is diminished when adjusting outcome for patient sociodemographic and anthropometric risk factors. For better transparency of data reporting from clinical quality registries the authors advocate dual reporting or risk stratified reporting of data.

## Conflict of interest

The authors declare the following financial interests/personal relationships which may be considered as potential competing interests: David Werner - Consultant Brainlab AG If there are other authors, they declare that they have no known competing financial interests or personal relationships that could have appeared to influence the work reported in this paper.
