# Supplementary material for: Beyond raw comparisons: Adjusted analysis reveals only minor inter-hospital differences in ACDF outcomes in Norway
Source: Brain Spine. 2026 Feb 16;6:105976. doi: 10.1016/j.bas.2026.105976 (PMC12933452; doi:10.1016/j.bas.2026.105976)
Supplement: Multimedia component 1 [file mmc1.docx]

Table 2. Logistic regression model predicting NDI raw score above 26 at 12 months after surgery. CI – 95% confidence intervals from the bootstrapped iterations.

| Predictor | Odds Ratio | 95% CI lower | 95% CI upper | Significance |
| --- | --- | --- | --- | --- |
| Age > 60 | 0.728 | 0.584 | 0.899 | 0.003 |
| Unemployed | 1.651 | 1.402 | 1.972 | 0.000 |
| Hard physical work | 1.267 | 1.058 | 1.528 | 0.012 |
| Lower educational level | 1.479 | 1.192 | 1.848 | 0.000 |
| Non native speaker | 1.746 | 1.296 | 2.349 | 0.000 |
| Litigation | 2.374 | 1.844 | 3.117 | 0.000 |
| Smoker | 1.339 | 1.120 | 1.613 | 0.002 |
| Previous cervical surgery | 2.095 | 1.647 | 2.683 | 0.000 |
| Duration arm pain 3 – 12 months | 1.832 | 1.418 | 2.450 | 0.000 |
| Duration arm pain > 12 months | 2.513 | 1.916 | 3.397 | 0.000 |
| Preop NDI 40-60 | 2.365 | 1.986 | 2.843 | 0.000 |
| Preop NDI >60 | 3.501 | 2.586 | 4.963 | 0.000 |
| Anxiety/Depression | 1.613 | 1.383 | 1.904 | 0.000 |
| NDI headache slight infrequently | 1.559 | 1.210 | 2.006 | 0.001 |
| NDI headache moderate infrequently | 1.824 | 1.423 | 2.366 | 0.000 |
| NDI headache moderate frequently | 2.941 | 2.237 | 3.935 | 0.000 |
| NDI headache severe frequently | 2.952 | 2.190 | 4.035 | 0.000 |
| NDI headache all the time | 4.390 | 3.053 | 6.560 | 0.000 |
| Private hospitals | 0.708 | 0.587 | 0.849 | 0.000 |
| Bergen university hospital | 1.109 | 0.896 | 1.365 | 0.335 |
| Oslo university hospital Rikshospitalet | 1.135 | 0.969 | 1.327 | 0.112 |
| Trondheim university hospital | 0.817 | 0.644 | 1.025 | 0.084 |
| Stavanger university hospital | 1.181 | 0.947 | 1.471 | 0.142 |
| Tromsø university hospital | 0.860 | 0.651 | 1.138 | 0.286 |
| Oslo university hospital Ullevaal | 1.35 | 0.796* | 2.290* | 0.266 |

Lower education – less than 4 years of university. Hard physical work – not working at a desk or with light physical labor. Anxiety/Depression as scored on the EQ-5D-5L form. Litigation – enrolled in litigation against the Norwegian medical welfare fund. Paresis – as scored based on patients perception. Compl. – complication at 12 months post surgery, as reported by the patient. NDI – Neck Disability Index. NRS – numeric rating scale. *CI from the parametric analysis due to the deviation function in SPSS.

Table 3. Logistic regression model predicting NRS arm score above 3 at 12 months after surgery. CI – 95% confidence intervals from the bootstrapped iterations.

| Predictor | Odds Ratio | 95% CI lower | 95% CI upper | Significance |
| --- | --- | --- | --- | --- |
| Age > 60 | .749 | 0.611 | 0.911 | 0.003 |
| Unemployed | 1.320 | 1.128 | 1.557 | 0.001 |
| Lower educational level | 1.359 | 1.115 | 1.672 | 0.003 |
| Non native speaker | 1.853 | 1.429 | 2.414 | 0.000 |
| Litigation | 1.595 | 1.254 | 2.035 | 0.000 |
| Smoker | 1.359 | 1.153 | 1.615 | 0.000 |
| Previous cervical surgery | 1.656 | 1.327 | 2.080 | 0.000 |
| Planned surgical level >1 | 1.187 | 1.007 | 1.402 | 0.037 |
| Duration arm pain 3 – 12 months | 1.875 | 1.464 | 2.475 | 0.000 |
| Duration arm pain > 12 months | 2.987 | 2.317 | 3.971 | 0.000 |
| Preop NRS arm pain 6-7 | 1.673 | 1.390 | 2.027 | 0.000 |
| Preop NRS arm pain >8 | 2.112 | 1.770 | 2.550 | 0.000 |
| Anxiety/Depression | 1.357 | 1.176 | 1.580 | 0.001 |
| NDI headache slight/moderate infrequently | 1.247 | 1.025 | 1.514 | 0.022 |
| NDI headache moderate frequently | 1.707 | 1.364 | 2.124 | 0.000 |
| NDI headache severe frequently | 1.652 | 1.308 | 2.099 | 0.000 |
| NDI headache all the time | 2.666 | 1.993 | 3.593 | 0.000 |
| Diabetes Mellitus II | 1.506 | 1.063 | 2.132 | 0.015 |
| Private hospitals | .725 | 0.614 | 0.857 | 0.000 |
| Bergen university hospital | 1.255 | 1.036 | 1.530 | 0.021 |
| Oslo university hospital Rikshospitalet | 1.016 | 0.874 | 1.176 | 0.834 |
| Trondheim university hospital | .889 | 0.710 | 1.105 | 0.291 |
| Stavanger university hospital | 1.155 | 0.931 | 1.422 | 0.178 |
| Tromsø university hospital | .902 | 0.684 | 1.170 | 0.437 |
| Oslo university hospital Ullevaal | 1.168 | 0.712* | 1.919* | 0.539 |

Lower education – less than 4 years of university. Hard physical work – not working at a desk or with light physical labor. Anxiety/Depression as scored on the EQ-5D-5L form. Litigation – enrolled in litigation against the Norwegian medical welfare fund. Paresis – as scored based on patients perception. Compl. – complication at 12 months post surgery, as reported by the patient. NDI – Neck Disability Index. NRS – numeric rating scale. *CI from the parametric analysis due to the deviation function in SPSS.

Table 4. Logistic regression model predicting NDI improvement by less than 35% 12 months after surgery. CI – 95% confidence intervals from the bootstrapped iterations.

| Predictor | Odds Ratio | 95% CI lower | 95% CI upper | Significance |
| --- | --- | --- | --- | --- |
| Age > 60 | 0.762 | 0.627 | 0.925 | 0.005 |
| Unemployed | 1.284 | 1.096 | 1.507 | 0.002 |
| Hard physical work | 1.191 | 1.003 | 1.415 | 0.044 |
| Lower educational level | 1.436 | 1.185 | 1.754 | 0.000 |
| Non native speaker | 1.782 | 1.366 | 2.373 | 0.000 |
| Litigation | 2.430 | 1.917 | 3.139 | 0.000 |
| Smoker | 1.224 | 1.025 | 1.458 | 0.019 |
| Previous cervical surgery | 2.018 | 1.614 | 2.529 | 0.000 |
| Planned surgical level >1 | 1.298 | 1.105 | 1.528 | 0.001 |
| Duration arm pain 3 – 12 months | 1.826 | 1.432 | 2.382 | 0.000 |
| Duration arm pain > 12 months | 2.681 | 2.100 | 3.543 | 0.000 |
| Preop NDI 40-60 | 0.729 | 0.610 | 0.866 | 0.000 |
| Preop NDI >60 | 0.651 | 0.477 | 0.872 | 0.007 |
| Anxiety/Depression | 1.445 | 1.250 | 1.679 | 0.000 |
| NDI headache slight infrequently | 1.438 | 1.146 | 1.799 | 0.001 |
| NDI headache moderate infrequently | 1.631 | 1.318 | 2.054 | 0.000 |
| NDI headache moderate frequently | 2.373 | 1.861 | 3.093 | 0.000 |
| NDI headache severe frequently | 2.164 | 1.654 | 2.915 | 0.000 |
| NDI headache all the time | 3.377 | 2.460 | 4.870 | 0.000 |
| Private hospitals | 0.711 | 0.604 | 0.831 | 0.000 |
| Bergen university hospital | 1.005 | 0.816 | 1.224 | 0.964 |
| Oslo university hospital Rikshospitalet | 1.163 | 1.003 | 1.350 | 0.143 |
| Trondheim university hospital | 0.853 | 0.683 | 1.053 | 0.143 |
| Stavanger university hospital | 1.203 | 0.984 | 1.481 | 0.078 |
| Tromsø university hospital | 0.905 | 0.702 | 1.161 | 0.444 |
| Oslo university hospital Ullevaal | 1.296 | 0.796* | 2.110* | 0.298 |

Lower education – less than 4 years of university. Hard physical work – not working at a desk or with light physical labor. Anxiety/Depression as scored on the EQ-5D-5L form. Litigation – enrolled in litigation against the Norwegian medical welfare fund. Paresis – as scored based on patients perception. Compl. – complication at 12 months post surgery, as reported by the patient. NDI – Neck Disability Index. NRS – numeric rating scale. *CI from the parametric analysis due to the deviation function in SPSS
